# Indoor Model Simulation for COVID-19 Transport and Exposure

**DOI:** 10.3390/ijerph18062927

**Published:** 2021-03-12

**Authors:** Tareq Hussein, Jakob Löndahl, Sara Thuresson, Malin Alsved, Afnan Al-Hunaiti, Kalle Saksela, Hazem Aqel, Heikki Junninen, Alexander Mahura, Markku Kulmala

**Affiliations:** 1 Department of Physics, The University of Jordan, Amman 11942, Jordan; 2 Institute for Atmospheric and Earth System Research (INAR/Physics), University of Helsinki, FI-00014 Helsinki, Finland; alexander.mahura@helsinki.fi (A.M.); markku.kulmala@helsinki.fi (M.K.); 3 Department of Design Sciences, Lund University, P.O. Box 118, SE-221 00 Lund, Sweden; jakob.londahl@design.lth.se (J.L.); sara.thuresson@design.lth.se (S.T.); malin.alsved@design.lth.se (M.A.); 4 Department of Chemistry, School of Science, University of Jordan, Amman 11942, Jordan; a.alhunaiti@ju.edu.jo; 5 Department of Virology, Helsinki University Hospital, University of Helsinki, FI-00014 Helsinki, Finland; kalle.saksela@helsinki.fi; 6 Department of Clinical Laboratory Sciences, Collage of Applied Medical Sciences, King Saud bin Abdulaziz University for Health Sciences, Jeddah 21423, Saudi Arabia; aqelh@ksau-hs.edu.sa; 7 Institute of Physics, Faculty of Science and Technology, University of Tartu, 51005 Tartu, Estonia; heikki.junninen@ut.ee; 8 Joint International Research Laboratory of Atmospheric and Earth System Sciences, School of Atmospheric Sciences, Nanjing University, Nanjing 210023, China; 9 Aerosol and Haze Laboratory, Beijing Advanced Innovation Center for Soft Matter Science and Engineering, Beijing University of Chemical Technology, Beijing 100029, China; 10 Faculty of Geography, Lomonosov Moscow State University, 119991 Moscow, Russia

**Keywords:** SARS-CoV-2, expiratory droplet, inhaled dose, indoor aerosol modeling

## Abstract

Transmission of respiratory viruses is a complex process involving emission, deposition in the airways, and infection. Inhalation is often the most relevant transmission mode in indoor environments. For severe acute respiratory syndrome coronavirus 2 (SARS-CoV-2), the risk of inhalation transmission is not yet fully understood. Here, we used an indoor aerosol model combined with a regional inhaled deposited dose model to examine the indoor transport of aerosols from an infected person with novel coronavirus disease (COVID-19) to a susceptible person and assess the potential inhaled dose rate of particles. Two scenarios with different ventilation rates were compared, as well as adult female versus male recipients. Assuming a source strength of 10 viruses/s, in a tightly closed room with poor ventilation (0.5 h^−1^), the respiratory tract deposited dose rate was 140–350 and 100–260 inhaled viruses/hour for males and females; respectively. With ventilation at 3 h^−1^ the dose rate was only 30–90 viruses/hour. Correcting for the half-life of SARS-CoV-2 in air, these numbers are reduced by a factor of 1.2–2.2 for poorly ventilated rooms and 1.1–1.4 for well-ventilated rooms. Combined with future determinations of virus emission rates, the size distribution of aerosols containing the virus, and the infectious dose, these results could play an important role in understanding the full picture of potential inhalation transmission in indoor environments.

## 1. Introduction

It has been evident that novel coronavirus disease (COVID-19), caused by the severe acute respiratory syndrome coronavirus 2 (SARS-CoV-2), is highly transmissible and infectious [1,2]. Due to its threat to global health, the WHO declared in March 2020 that the COVID-19 epidemic should be considered a global public health emergency.

Being at a close distance (within 1–2 m) with an individual with COVID-19 (either symptomatic or asymptomatic), especially in an environment with poor ventilation, has been associated with a high risk for COVID-19 transmission, even when individuals take care not to touch. In general, respiratory virus transmission can occur via two main possible pathways [3,4,5,6,7,8,9,10,11]:

1. Contact spread either by the direct pathway or indirect (i.e., contaminated surface) pathway;
2. Transmission by the inhalation of aerosols and droplets either at short range (commonly known as droplet transmission) or long range (commonly known as airborne transmission).

Contact spread requires susceptible individuals to physically touch a virus-contaminated hand (e.g., a handshake with a person with the virus) or touch a contaminated surface (e.g., a handle or handrail). Transmission by inhalation may occur without physical contact between carriers and susceptible individuals. It is of special concern in indoor environments where people spend long periods of time together, such as homes, offices, restaurants, or gyms. The risk of disease transmission through inhalation increases if ventilation is poor and when people speak or sing loudly. Eventually, infectious airborne particles settle and deposit onto surfaces and might become transmissible and infect a susceptible person via indirect contact [6,12].

Coronavirus is believed to be affected by ambient conditions. Therefore, its survival in aerosols as well as on different surfaces depends on the air temperature, relative humidity, UV radiation, material that embeds the virus, reactive atmospheric molecules (such as ozone), and the type and pretreatment of surfaces [12,13,14,15,16].

During the COVID-19 pandemic, public health awareness has increased on measures to reduce disease transmission, such as washing hands, avoid handshaking, keeping distance, using masks, and improving ventilation. There are, however, still major knowledge gaps on the characteristics of aerosols and droplets containing SARS-CoV-2 and their concentration patterns and behavior in various environments. This is partly due to the difficulties in sampling virus-laden aerosols, as concentration levels often are very low (i.e., below the detection limit).

Estimating the actual probability of transmission through inhalation requires information from the virology and medicine as well as aerosol sciences. Virology provides information about things such as viral titer in the respiratory fluid, viability of the viruses during various types of stress, and functional molecular structures for the virus. Medical knowledge is necessary to assess the immune response, clearance mechanisms, and infectious dose for different transmission pathways. Aerosol science provides information about how the infectious particles move through the air to a susceptible individual. Neither the aerosol viral load nor the minimum infectious dose for COVID-19 have been definitively determined, as human challenge tests have not yet been performed. Emitted viral loads from exhaled air depend on a number of factors, including the concentration of the virus in saliva and lung-lining fluid, type of vocal or respiratory activities, and ventilation rates. Current estimates of infectious doses, based on modeling and animal studies, indicate that the number of viable virions needed to cause a disease are to the order of 10–1000 or more [17,18,19,20,21,22]. Besides that, it is also necessary to calculate how COVID-19 infectious particles are transported and dispersed through the air to a susceptible individual and deposited in the human respiratory system. The combination of an infectious dose, the viral load in emitted particles, airborne transport, and respiratory tract deposition can help paint a wider picture of inhalation transmission indoors.

The objective of this study was to develop a model for estimation of the transport and regional respiratory tract deposition of the airborne SARS-CoV-2 virus in indoor environments. Although a number of exposure models are available for the indoor transmission of pathogens, these do not generally include respiratory tract deposition of aerosols or breathing flow rates. These are critical parameters for determining the dose of particles that actually reaches specific targets in the body. As an example, the model was used to compare two types of indoor environments for men and women with and without virus inactivation. The required information for the model simulations was obtained from a literature review about the physical characteristics of COVID-19-related aerosols.

## 2. Materials and Methods

### 2.1. Model Overview

We utilized an indoor aerosols model combined with a regional respiratory tract deposited dose model that take activity patterns and gender into account. Constant values were used for the virus emission rates and half-life of the virus viability from environmental stress. Transmission by inhalation was conceptualized as follows:

1. During respiratory activities, such as talking, singing, coughing, or sneezing, aerosols and droplets of virus-laden respiratory tract fluid are emitted into the surrounding air;
2. These droplets are subject to evaporation and thus shrink in size and become dry particles (hereafter, the infectious particles). Some will become small enough to remain airborne for minutes to hours. SARS-CoV-2 may remain infectious in such particles over these time scales;
3. A susceptible person inhales the infectious particles.

Gas flow velocities varied with the type of exhalation (e.g., processes of breathing, talking, coughing, sneezing, or singing) [10,11] and thereby affected how far the particles spread in the initial phase. However, the small particles (roughly <5–10 µm) that may remain airborne for hours typically spread within the size of a room over time by following air currents caused by ventilation, drafts, and movements.

### 2.2. Formation and Emission of SARS-CoV-2 Aerosols: Physical Characterisitics

Similar to many respiratory diseases (such as influenza, tuberculosis, and measles), SARS-CoV-2 is emitted during vocal and respiratory activities. By number, exhaled particles during breathing are primarily below 5 µm and have been demonstrated to originate from the lower rather than the upper respiratory tract [23]. During talking or singing, larger particles are also emitted from the upper airways [24]. Four mechanisms describe their formation as airborne particles [10,25,26]:

1. Open-close cycling of airway structures in the distal lung;
2. Open-close cycling of glottic structures, primarily during talking and singing;
3. Shear forces due to high-velocity gas flow, primarily in the upper respiratory tract;
4. Articulation of consonants, generating saliva particles from the oral cavity.

Gas flow velocities vary with the type of exhalation [10,11]:

1. Tidal volume breathing generates airflow velocities around 1 m/s in the trachea and bronchi;
2. Talking generates airflow velocities up to 5 m/s at the initial or starting phase of the motion;
3. Coughing generates airflow velocities between 2–50 m/s;
4. Sneezing generates airflow velocities more than 100 m/s.

The source strength of exhaled SARS-CoV-2 is the largest uncertainty in the calculations, as it is presently incompletely investigated and highly fluctuating. Both the inter and intrasubject variability are high due to factors such as days since symptom onset, type of vocal activity, viral loads in the respiratory tract, and breathing patterns. Often, emission rates are provided in quanta per unit of time rather than the number of virus particles, where inhalation of a quantum is the amount of pathogen needed to cause infection in 63% of the population on average [27]. As previously described, for SARS-CoV-2, a quanta was estimated to correspond to 10–1000 viruses or more [17,18,19,20,21,22]. In order to provide a more direct link to studies measuring the virus concentration in the respiratory tract lining fluids and exhaled particle concentrations, we chose to describe the emission rate by an absolute number of 10 viable viruses per second rather than in terms of quanta. This corresponded to a source strength to the order of 10–1000 quanta/hour, which was the typical range reported in the literature for COVID-19 [19].

In addition, the diameter of the droplet relative to the dry particle (D_p_*drop*_*/D_p_dry_*) relation varies with the respiratory liquid components (salts, proteins, and surfactants), which may change dramatically during an infection [28].

### 2.3. Exposure to Airborne Particles Containing SARS-CoV-2

#### 2.3.1. Indoor Aerosol Model

The indoor aerosol model (IAM) has several important applications, where one is human exposure assessment, which provides information about the real-time exposure level and the deposited dose in the respiratory system [29]. The amount of aerosols presented in indoor air is governed by the source strength, transformation processes, and deposition of aerosol particles.

The indoor aerosols can be of an indoor or outdoor origin. However, in the case of airborne SARS-CoV-2, indoor aerosol sources are the ones of major concern, and outdoor air could be considered to dilute the indoor concentrations of infectious aerosols. The state and properties of indoor aerosols are modified by changes in the ambient conditions (e.g., air temperature and relative humidity). Eventually, indoor aerosols are either deposited onto surfaces or removed from the indoor air via air cleaners or ventilation. Aerosol particles also undergo complex processes through aerosol dynamics and chemical reactions that change their state, concentration, and physical–chemical properties. This dynamic behavior of indoor aerosols can be described by the mass balance equation [30,31], which is a first-order differential equation. The simple IAM describes the dynamic behavior of a single component (e.g., total aerosol particle number concentration) inside a single compartment. Mathematically, it is written as

(1) $$\frac{dI}{dt}=P\lambda O-\left(\lambda +\lambda_{d}\right)I+S_{in}$$

where *t* is the time, *I* and *O* are the indoor and outdoor concentrations of the aerosol particles, respectively, *P* is the penetration factor of aerosol particles while being transported from the outdoor air into the indoor air, *λ* is the ventilation rate, *λ_d_* is the deposition rate of aerosol particles onto available indoor surfaces, and *S_in_* represents the emission rates from an indoor source. Well-mixed indoor air is a key assumption for the mass balance equation to be valid [30]. Otherwise, spatial variation of indoor aerosol particle concentrations must be taken into account by, for example, utilizing computational fluid dynamic (CFD) models.

In general, the mass balance equation (Equation (1)) can be solved numerically, but it holds an analytical solution whenever *O*, *P*, *λ*, *λ_d_*, and *S_in_* are all constant in time:

(2) $$I\left(t\right)=I_{0}e^{-\left(\lambda +\lambda_{d}\right)t}+\frac{P\lambda O+S_{in}}{\lambda +\lambda_{d}}\left[1-e^{-\left(\lambda +\lambda_{d}\right)t}\right]$$

where *I*(*t*) is the number concentration of indoor aerosols, *I*_0_ is the initial concentration of indoor aerosols at *t* = 0, and all other parameters were defined right after Equation (1). A constructive sensitivity analysis for this simple IAM is presented and summarized in Appendix A.

#### 2.3.2. Inhaled Deposited Dose Model

A key link to relate indoor air quality to the biological response is the inhaled deposited dose. It is defined as the amount of aerosol deposited in the respiratory tract during breathing for a certain time period. As described by Hussein et al. [29], the inhaled deposited dose can be expressed as

(3) $$D=\overset{t_{2}}{\underset{t_{1}}{\int }}\overset{D_{p2}}{\underset{D_{p1}}{\int }}V_{E}\cdot DF\cdot n_{N}^{0}\cdot d\log D_{p}dt$$

where *V_E_* is the minute ventilation (or breathing rate, as the volume of air breathed per time interval), *DF* is the deposition fraction of aerosol particles in the respiratory system, and $n_{N}^{0}$ = *dN*/*d*log(*D_p_*) is the lognormal particle number size distribution. Both *DF* and $n_{N}^{0}$ are functions of log(*D_p_*) where *D_p_* is the particle diameter. The time integral is evaluated for an exposure time period Δ*t* = *t*_2_ − *t*_1_ based on any selected time step.

To simplify the situations, we can consider a certain particle type (i.e., size and shape) and calculate the dose rate (i.e., deposited particles per hour):

(4) $$DR=V_{E}\cdot DF\cdot I$$

**Figure 1:**
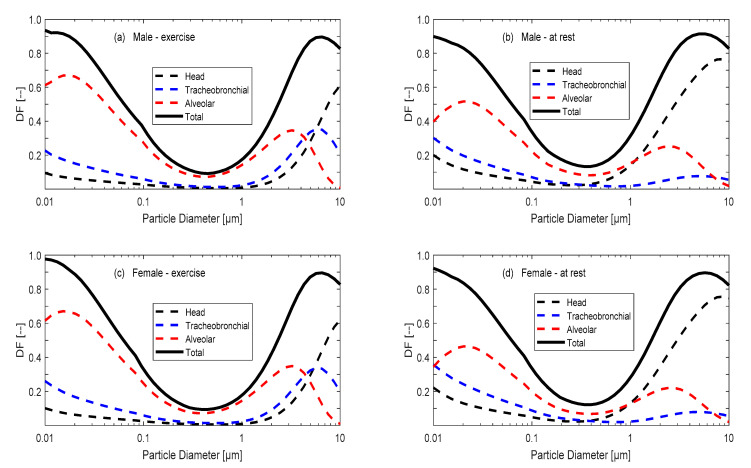
Size-resolved deposition fraction (*DF*) curves inside the respiratory tracts of the following adults: (**a**) males exercising, (**b**) males at rest, (**c**) females exercising, and (**d**) females at rest. Data was adopted from Löndahl et al. [35] and the International Commission on Radiological Protection (ICRP) and Multiple-Path Model of Particle Deposition (MPPD) models.

The minute ventilation (*V_E_*) depends on the body size of the person, gender, age, health status, and the physical activity of the person (Table 1) [32,33,34]. The deposition fraction (*DF*) varies with different parts of the respiratory system (head and throat (H), tracheobronchial (TB), and pulmonary and alveolar (Alv) (see, for example, Figure 1), according to Löndahl et al. [35]. The indoor aerosol concentrations (*I*) can be taken from the indoor aerosol model simulation at steady state conditions (i.e., *I_steady_*).

## 3. Results

### 3.1. Scenarios of Expiratory Airborne Particles Indoors: Exposure Levels

**Figure 2:**
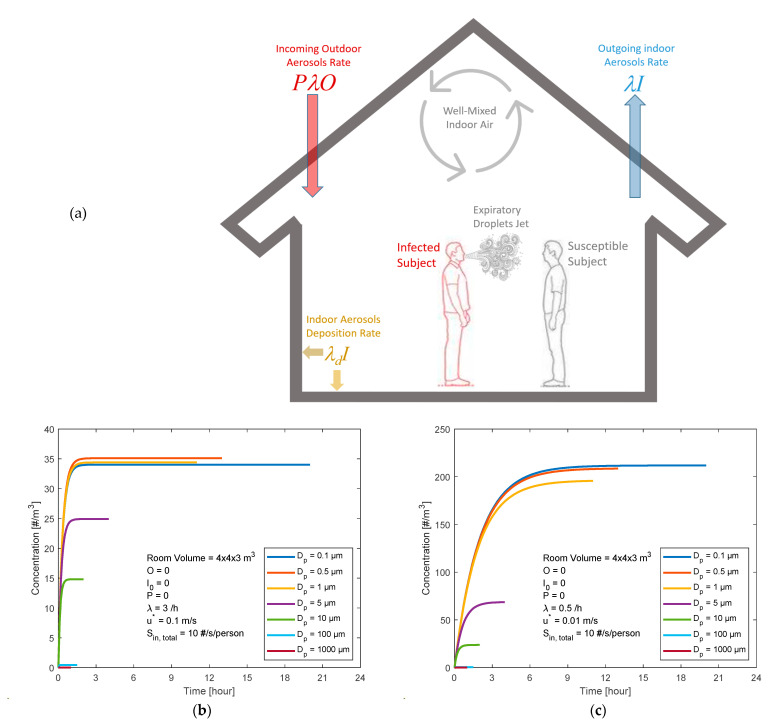
A room occupied by an infected person emitting expiratory particles. (**a**) A scenario sketch and the indoor infectious virus particles concentrations as a function of their diameter and of time for (**b**) a well-ventilated room (high ventilation rate *λ* = 3 h^−1^ and low friction velocity u* = 0.1 m/s) and (**c**) a tightly closed room (low ventilation rate *λ* = 0.5 h^−1^ and low friction velocity u* = 0.01 m/s). The total number of emitted viruses (Sin, total = 10 viruses/s) was assumed to be uniformly distributed on the particles, with an equal number for all particle sizes (7 size bins in the range of 0.1–1000 µm).

We start with a scenario with the following assumptions (Figure 2a):

▪ A room (4 × 4 × 3 m^3^) with well-mixed indoor air;
▪ A clean (not contaminated) room at *t* = 0 (i.e., airborne particles containing SARS-CoV-2 concentrations indoors *I*_0_ = 0 m^−3^);
▪ Clean outdoor air (i.e., airborne particles containing SARS-CoV-2 concentrations outdoors *O* = 0 m^−3^);
▪ The penetration factor (*P*) was set to zero;
▪ Ventilation rate (*λ*) varying within the range of 0.5–3 h^−1^;
  - Well-ventilated indoor air (i.e., high ventilation rate *λ* = 3 h^−1^ and low friction velocity u* = 0.1 m/s);
  - Poorly ventilated indoor air (i.e., low ventilation rate *λ* = 0.5 h^−1^ and low friction velocity u* = 0.01 m/s);
▪ The deposition rate (*λ_d_*) depends on the particle diameter (*D_p_*) and the turbulent mixing conditions (i.e., friction velocity u* in the range 0.01–0.1 m/s);
▪ Occupancy by a person with the virus who emits expiratory airborne particles carrying SARS-CoV-2 virions;
▪ The expiratory airborne particles of relevance for transmission of COVID-19 through inhalation have particle diameters (*D_p_*) within the range of 0.1–1000 µm;
▪ Emission rates may vary by many orders of magnitude, depending on the characteristics of the source. Here, as an example, we use a rate of 10 viruses/second;
  - The viruses are assumed to be uniformly distributed on the particles, with an equal number for all particle sizes and with 7 size bins in the range of 0.1–1000 µm (i.e., ~1.4 viruses/second in each particle size bin);
  - The true size distribution is not known, but large particles will contain more viruses while, on the other hand, there are many small particles by number;
  - Thus, the approximation of a uniform distribution of viruses over all sizes is in fact in reasonable agreement with the few measurements that exist [6,36,37].

We evaluated the indoor aerosol model simulation for two conditions: a well-ventilated room and a tightly closed (poorly ventilated) room, presented in Figure 2b,c. The *I_steady_* of the supermicron (*D_p_* > 100 µm) particles was less than 3 m^−3^ for both the well-ventilated and nonventilated conditions.

When the room was well-ventilated (i.e., high ventilation rate *λ* = 3 h^−1^ and low friction velocity u* = 0.1 m/s), the indoor concentration of exhaled infectious particles reached its steady state (*I_steady_*) levels within about 1.5 h after the infected person entered the room. For the submicron fraction (*D_p_* < 1 µm), the infectious particles *I_steady_* exceeded 33 m^−3^. The micron particles (5 and 10 µm) reached an *I_steady_* of ~25 and 15 m^−3^, respectively.

For the poorly ventilated room (i.e., low ventilation rate *λ* = 0.5 h^−1^ and low friction velocity u* = 0.01 m/s), the submicron fraction of infectious particles *I_steady_* exceeded 190 m^−3^ after 9 h. The micron particles (5 and 10 µm) reached an *I_steady_* of ~69 and 24 m^−3^, respectively.

### 3.2. Scenarios of COVID-19 Exposure: Inhaled Deposited Dose Rate

The susceptible exposure scenarios were followed after the calculations for *I_steady_* illustrated in the previous section (Figure 2a). As shown in Figure 1, the total *DF* curve was rather similar for men and women, and it was also similar for the activity status, such as resting or exercising. Therefore, we interpolated the *DF* total curve for the particle diameters used in the exposure levels model simulation (i.e., *D_p_* = 0.1, 0.5, 1, 5, and 10 µm). We dropped out the supermicron particle cases (i.e., *D_p_* = 100 and 1000 µm) because of negligible contribution.

We further simplified the minute ventilation (*V_E_*) assumptions to consider resting (e.g., sitting or standing) or exercising (e.g., light office or medical work) activities. Approximately, the *V_E_* assumed for an adult female was 0.45 and 1.15 m^3^/h during resting and exercising, respectively. For an adult male, the *V_E_* assumed was 0.63 and 1.56 m^3^/h, respectively.

Recalling the exposure levels presented in Figure 2b,c, the calculated inhaled deposited dose rates of the expiratory infectious particles are listed in Table 2. Obviously, the dose rate for males was higher because the *V_E_* was higher for males than females. The dose rate was higher for the nonventilated room than the well-ventilated room conditions. This was basically because the *I_steady_* was higher for the nonventilated room than the well-ventilated room conditions. In addition, the dose rate was higher during the exercising activities than the resting activities.

Regarding the particle size, the lowest dose rate by number was for the 10 µm particles, and the highest was for particles with a diameter = 0.1 µm. However, the micron particles were expected to carry more SARS-CoV-2 virions than the submicron particles, based on their larger volume. As a simple example, we may assume that respiratory droplets shrink to half the diameter size in ambient air, that the infected patient has a viral load of 10^9^ virus mL^−1^, and that the virions are uniformly distributed in the exhaled aerosol. Then, the 10 µm particles would contain 4 virions each, 50% of the 5 µm particle would contain one virion, and 3% of the 1 µm particles would contain one virion. Only a very small number of submicrometer particles would contain a virus. On the other hand, the smaller particles were usually higher in number than the larger ones, and as previously explained, it was a reasonable approximation that there were equal numbers of virions in the size bins. The theoretical model calculations suggest that the deposited dose after spending an hour in a room together with a person with COVID-19 could be around 100 or 350 viruses at rest and at exercise, respectively, in a tightly closed room and around 30 or 90 viruses at rest and at exercise, respectively, in a well-ventilated room.

According to van Doremalen et al. [12], the median estimate for the half-life of SARS-CoV-2 in aerosols was about 1.1 h, although this depended highly on the ambient conditions. Taking that into consideration in the IAM and the selected exposure scenario, the infectious aerosol particle concentrations were reduced by a factor of 1.2–2.2 for the tightly closed room conditions (e.g., dominating in wintertime) and a factor of 1.1–1.4 for the well-ventilated room conditions (e.g., summertime).

## 4. Discussion

Similar to many respiratory diseases (such as influenza, tuberculosis, and measles), COVID-19 has been shown to transmit efficiently through inhalation, especially in confined spaces with poor ventilation and long residence times from people [38,39,40,41,42,43,44,45,46,47,48,49,50]. In order to limit inhalation transmission of SARS-CoV-2 indoors, it has been confirmed that room ventilation, natural ventilation, avoiding air recirculation, open space, proper use and disinfection of the premises, sanitization of surfaces, and avoiding crowds gathering with asymptomatic carriers can be considered as effective protocols [3,6].

The indoor transport mechanisms of aerosols are complicated and require careful understanding about the air status, such as stagnant versus stirred air. Both experimental and modeling investigations are needed to understand the transport in the form of an aerosol cloud or a single particle trajectory. In this study, we applied an indoor exposure model to determine the concentration of aerosol particles in indoor environments and the deposited dose following inhalation of these particles. In addition, we compared a number of transmission scenarios based on the ventilation in the room and basic characteristics of the exposed persons.

A critical but so far largely unknown number is the virus emission rate, which will differ by many orders of magnitude depending on the individual that is the source. Only a single virus emission rate was used in the calculations presented here, as this number still is poorly characterized from experiments and will differ by many orders of magnitude depending on the individual that is the source. For instance, the viral load in the upper respiratory tract may vary between at least 10^3^–10^10^ RNA copies/mL for moderate to severely ill patients during the first few days from symptom onset [51,52]. In addition to this, the number of exhaled aerosol particles varies by 2–3 orders of magnitude between breathing, normal talking, and shouting or loud singing and even more for coughing [24,53]. In addition, the duration of these activities matters. Breathing may, for instance, sometimes shed more of the virus than coughing because it is continuous, and coughs are less frequent [54]. Some individuals also emit substantially higher amounts of particles than average [55,56].

Furthermore, we still have a poor understanding of the size distribution of aerosol particles that carry viable viruses. The relationship between the particle size and viral load is complex and poorly investigated. Large particles typically originate from the mouth and smaller particles from the lower airways. Both types may be produced during common activities such as talking or just breathing. The viral load in saliva (from the mouth) and sputum (from lower airways) differs between patients, in part depending on the primary site of infection. A person with a primary infection in the lower airways, thus having a higher viral load in this region, could produce respiratory droplets and aerosols where the smaller sized particles have higher viral loads than the large particles. In those cases, a high dose rate of small particles could pose an increased risk of infection. Particles below five micrometers readily reach the whole respiratory tract once inhaled [57]. According to the collection of deposited expiratory droplets (size distribution in a diameter range of 0–1000 µm) on glass slides in front of a person, the peak diameter was in the diameter range of 8–100 µm during talking or coughing [9,53,54]. For most types of aerosol emissions, smaller particles are more numerous than larger ones. Nevertheless, the fewer larger particles may carry a substantial part of the emitted mass due to their larger volume. Taken together, it is a tolerable guess that the viruses are distributed relatively uniformly over the whole aerosol particle size range, as we presumed in the present calculations. This is also supported by the limited experimental data available [6,36,37]. An interesting question is: how pre- or asymptomatic infected individuals generate aerosols without coughing or sneezing? In fact, ordinary breathing and speech both emit numerous quantities of aerosol particles [58,59,60,61,62,63].

The total number of airborne expiratory particles increased from about 3 × 10^6^ m^3^ to about 7 × 10^6^ m^3^ right in front of the person talking or coughing [9]. Liu et al. [6] reported that the SARS-CoV-2 airborne concentration existed in two main peaks within the diameter ranges of 0.25–1 µm (49 copies/m^3^) and >2.5 µm (7–9 copies/m^3^). The deposition rate of expiratory droplets containing SARS-CoV-2 was in the range of 31–113 copies/m^3^/hour.

The model we used was limited, as it was based on the steady state concentrations in a well-mixed room. In reality, when two persons are talking to each other, they are likely within a 2 m distance, and the person without the virus would then be exposed to higher aerosol concentrations than reported here. However, our results could be interpreted as the minimum dose for anybody present in the room together with a person who emits relatively high numbers of viruses. For instance, the Wells–Riley model of transmission assumes that the air in a room is well-mixed [11,38]. However, exhaled particles (either indoors or outdoors) transport in a puff or plume that travels in the direction of the background air motion [10]. Making the situations more complicated, increased air speeds during coughing or sneezing might serve to transport the expiratory particles further and reach additional susceptible people. In contrast, air turbulence can dilute the particle concentration and reduce the chance of infection. Furthermore, droplets and expiratory particles may settle fast enough thanks to gravity to be removed from the air before being inhaled.

Coronaviruses are affected by ambient conditions. Its survival in aerosols, droplets, and on surfaces depends on the air temperature, relative humidity, radiation, reactive species in the air, and the type and pretreatment of surfaces [12,13,14,15,16]. For example, Casanova et al. [13] showed that the infectious virus persisted on stainless steel surfaces for as long as 28 days when the air temperature was 4 °C, and the lowest level of inactivation occurred at 20% relative humidity. The higher the air temperature is, the shorter the virus lifetime will be on surfaces as well as in aerosols [15,16]. In fact, the combination of temperature and relative humidity plays a major role in SARS-CoV-2 survival. For example, the virus infectivity generally decreases in conditions with a high relative humidity and temperature and increases with a low relative humidity and low temperature (although the relationship with humidity is somewhat more complex) [64,65].

In general, there is little information on the characteristics of airborne SARS-CoV-2-containing aerosols, their concentration patterns, and their behavior during airborne transmission due to the difficulties in sampling virus-laden aerosols and challenges in their quantification at low concentrations.

The transmission of respiratory viruses is a complex process involving emission, deposition in airways, and the infection of host cells. Inhalation is often the most relevant transmission mode in indoor settings, especially during low air ventilation settings. For SARS-CoV-2, the risk of inhalation transmission is not yet fully understood.

## 5. Conclusions

In this study, we utilized a simple indoor aerosol model combined with a regional inhaled deposited dose model to examine the indoor transport of aerosols from a person with COVID-19 to a susceptible person and assessed the potential inhaled dose rate of particles in the respiratory tracts. Two scenarios with different ventilation rates were compared, as well as adult female versus male recipients.

The results from our model investigations showed that in a tightly closed room with poor ventilation (0.5 h^−1^), the inhaled dose rate was 140–350 particles/hour for males and 100–260 particles/hour for females. With enhanced ventilation settings (3 h^−1^), the dose rate was 40–90 particles/hour and 30–70 particles/hour, respectively. The variation in these numbers can be explained by the inhaled particle size, gender, and person’s activity. In the poor air ventilation setting, the maximum was obtained for submicron particles during exercise activities. When the air ventilation setting was improved (e.g., well-ventilated), the maximum was also obtained during exercise activities, but the inhaled dose rate for micron particles was higher than that for submicron particles. In general, the dose rate for females was higher than that for males by a factor of 1.3–2. Correcting for the half-life of SARS-CoV-2 in air, these dose rates were reduced by a factor of 1.2–2.2 for poorly ventilated rooms and 1.1–1.4 for well-ventilated rooms.

As described, there are still major knowledge gaps that make the model calculations highly uncertain. Nevertheless, model calculations like these make it possible to relatively easily approximate how the inhaled amount of viruses is affected by factors such as room ventilation, breathing flow rate, gender, room size, aerosol size distribution, exposure time, level of exercise, and the type of vocal activity. Combined with future determinations of the infectious dose and viral load of emitted droplets and aerosols of infectious individuals, these results could play an important role in understanding the full picture of potential inhalation transmission in indoor environments.

## Figures and Tables

**Table 1:** Minute ventilation (volume of air breathed), *V_E_* (m^3^/h), for adults according to Holmes [32]. The last column indicates the deposition fraction (*DF*) curve used for that activity (Figure 2).

Activity	Female	Male	*DF* Curve Type
Yard work	1.08	1.74	Exercise
Walking (4.0 km/h)	1.20	1.38	Exercise
Standing	0.48	0.66	At rest
Sitting	0.42	0.54	At rest

**Table 2:** Dose rate (viruses/hour) as a function of the particle diameter (µm), room conditions, human gender, and physical activity for the exposure levels illustrated in Figure 2b,c. Here, *V_E_* (m^3^/h) is the minute ventilation (volume of air breathed) and *DF* is the deposition fraction in the respiratory system.

Room Conditions			*V_E_*	0.1 µm	0.5 µm	1 µm	5 µm	10 µm	Total
		*DF*		0.34	0.12	0.24	0.89	0.83	
Tightly Closed (ventilation = 0.5 h^−1^)	Male	Rest	0.63	46	16	30	39	13	143
		Exercise	1.56	114	40	74	94	31	354
	Female	Rest	0.45	33	11	21	27	9	101
		Exercise	1.15	84	29	54	70	23	260
Well-Ventilated (ventilation = 3 h^−1^)	Male	Rest	0.63	7	3	6	14	9	39
		Exercise	1.56	19	7	13	36	20	94
	Female	Rest	0.45	6	1	4	10	6	27
		Exercise	1.15	14	4	10	26	14	69

**Figure A1:**
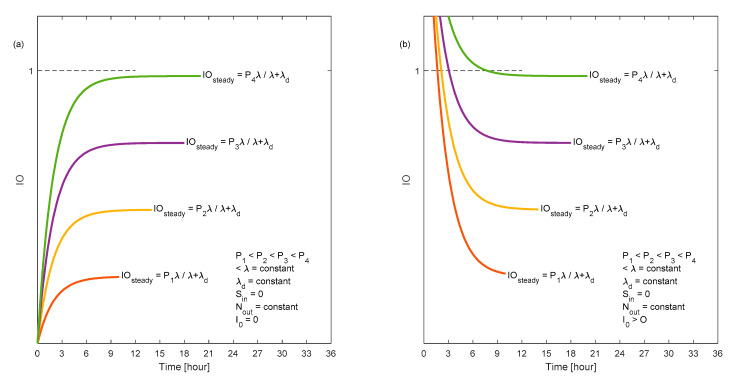
Role of the penetration factor (*P*) on the steady state indoor aerosol concentration (i.e., steady state indoor-to-outdoor concentration *IO_steady_*) (**a**) when the initial indoor aerosol concentration *I*_0_ = 0 and (**b**) when the initial indoor aerosol concentration is higher than that outdoors (i.e., *I*_0_ > O). All other indoor aerosol parameters are held constant, as shown on the subplots.

**Figure A2:**
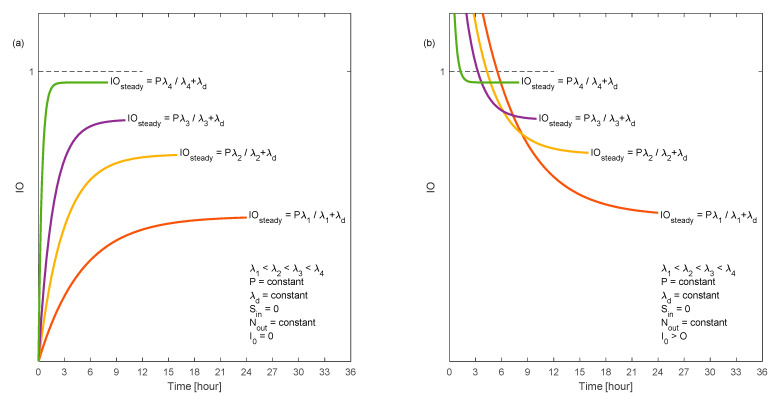
Role of the ventilation rate (*λ*) on the steady state indoor aerosol concentration (i.e., steady state indoor-to-outdoor concentration *IO_steady_*) (**a**) when the initial indoor aerosol concentration *I*_0_ = 0 and (**b**) when the initial indoor aerosol concentration is higher than that outdoors (i.e., *I*_0_ > O). All other indoor aerosol parameters are held constant, as shown on the subplots.

**Figure A3:**
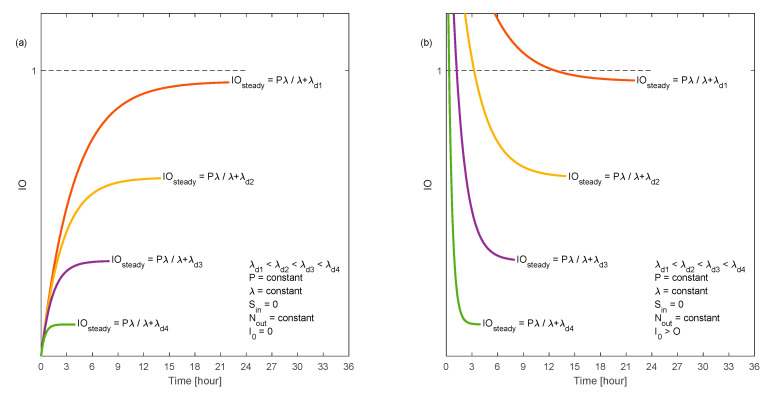
Role of the ventilation rate (*λ*) on the steady state indoor aerosol concentration (i.e., steady state indoor-to-outdoor concentration *IO_steady_*) (**a**) when the initial indoor aerosol concentration *I*_0_ = 0 and (**b**) when the initial indoor aerosol concentration is higher than that outdoors (i.e., *I*_0_ > O). All other indoor aerosol parameters are held constant, as shown on the subplots.

**Figure A4:**
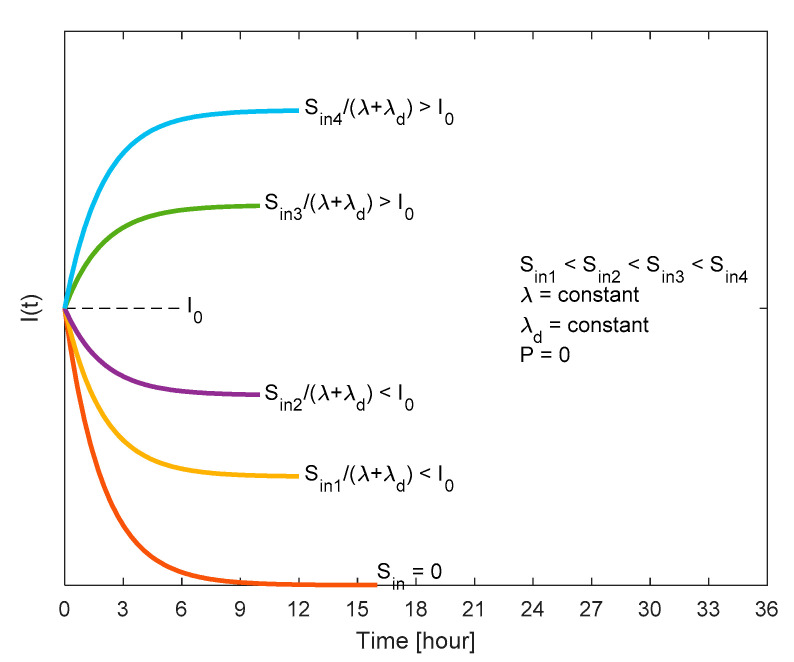
Role of the ventilation rate (*S_in_*) on the steady state indoor aerosol concentration. All other indoor aerosol parameters—*λ*, *λ_d_*, and *P—*are held constant as shown in the figure.

## Appendix A. Sensitivity Analysis for the Simple Indoor Aerosol Model (IAM)

In this study, the sensitivity analysis of the indoor aerosol model (IAM) was tested to illustrate the role and contribution of the model parameters (*O*, *P*, *λ*, *λ_d_*, and *S_in_*) on the time evolution of the indoor aerosol concentrations reaching the steady state. The most important part was how long it took to reach a steady state concentration (*I_steady_*). Sometimes, it is valuable to explore the indoor-to-outdoor concentration (*IO*) ratio when relating the time evolution of the indoor aerosol concentrations to those outdoors, where *O* is constant. The sensitivity analysis was performed for a single room (4 × 4 × 3 m^3^) with the following assumptions:

1. Empty room;
2. Unoccupied;
3. Well-mixed indoor air;
4. Constant model parameters (*O*, *P*, *λ*, *λ_d_*, and *S_in_*).

Firstly, the role of the penetration factor (*P*) was assessed when the initial indoor concentration was zero (i.e., *I*_0_ = 0) and the outdoor aerosol concentration was constant (i.e., *O* were arbitrary and constant). The steady state value of the *IO* ratio (i.e., *IO_steady_*) was achieved after a certain period of time (Figure A1a). The higher the value of *P*, the higher the *IO_steady_* value will be. Recalling the analytical solution of the mass balance equation, the *IO_steady_* = *P*/(*λ* + *λ_d_*). When the initial indoor aerosols concentration was higher than that outdoors (i.e., *I*_0_ > *O*), the steady state conditions (*IO_steady_* = *P*/(*λ* + *λ_d_*)) was also reached after the same time as in the first case (Figure A1b). The time at which the steady state condition was achieved depended on the ventilation rate, as will be illustrated in the second part of the sensitivity analysis.

Secondly, the role of the ventilation rate (*λ*) was assessed when *I*_0_ = 0 or *I*_0_ > *O*. For both cases, we considered *O* as constant (Figure A2). Again, the *IO_steady_* = *P*/(*λ* + *λ_d_*) was achieved regardless of the initial value of the indoor aerosols concentration (*I*_0_). The higher the *λ* was, the higher the *IO_steady_* was, and the less time was needed to achieve the *IO_steady_* value. Interestingly, when *λ* >> *λ_d_*, the *IO_steady_* = *P*, which usually occurs for aerosol particles with diameters in the range of 0.1–1 µm.

Thirdly, the role of the deposition rate (*λ_d_*) behaved in a similar way (Figure A3). As expected, the *IO_steady_* was achieved regardless to the initial value of the indoor aerosols concentration (*I*_0_). The higher the *λ_d_* was, the lower the *IO_steady_* was, and the shorter the time needed to achieve the *IO_steady_* value (i.e., *P*/(*λ* + *λ_d_*)).

Fourthly, the role of the indoor source strength (*S_in_*) was tested by assuming that the indoor aerosols originated from indoor sources only (i.e., *P* = 0 and *S_in_* ≠ 0) (Figure A4). Again, the *I_steady_* was achieved regardless of the initial value of the indoor aerosol concentration (*I*_0_). The higher the *S_in_* was, the higher the *I_steady_* was. In this case, the *I_steady_* = *S_in_*/(*λ* + *λ_d_*)). Here, the source term (*S_in_*) in the mass balance equation competes against the aerosol particle losses (–(*λ* + *λ_d_*)*I*). When *S_in_* > *λ* + *λ_d_*)*I*, the indoor aerosol concentrations grow, reaching its steady state value (*I_steady_*). When *S_in_* < (*λ* + *λ_d_*)*I*, the indoor aerosol concentration decays, reaching its steady state value (*I_steady_*). Both the growth rate and the decay rate of the indoor aerosol concentrations depend on the total loss rate (i.e., *λ* + *λ_d_*), and *I_steady_* is inversely proportional to *λ* + *λ_d_*.
